# Racism in German healthcare: uncovering the construction and silencing of the “other”

**DOI:** 10.3389/fpubh.2024.1485933

**Published:** 2025-01-07

**Authors:** Tanja Gangarova, Melike Yildiz, Lina Kabangu

**Affiliations:** ^1^National Discrimination and Racism Monitor (NaDiRa), German Center for Integration and Migration Research (DeZIM), Berlin, Germany; ^2^Familia+Migra Network, Berlin, Germany; ^3^CSE Caritas, Essen, Germany

**Keywords:** racism, othering, silencing, CBPR, healthcare, Germany

## Abstract

While the impact of racism on healthcare interactions has been researched extensively in many parts of the world, substantive studies on healthcare-related racism in Europe, and particularly in Germany, remain scarce. This paper builds on a study that applies Community-Based Participatory Research (CBPR) and aims to explore healthcare users’ experiences of racism within German healthcare. Community members were trained as peer researchers and given support as they conducted a total of six focus group discussions that involved a total of 14 study participants: these participants were organized into two subsamples of seven participants each (subsample one: Black, African, Afro-diasporic healthcare users; subsample two: healthcare users perceived or self-describing as Muslim), and each subsample had three focus group discussions. A democratic approach to qualitative data analysis was applied in the form of the DEPICT model. The data analysis developed iteratively, with inductive and deductive steps complementing one another. The study results illustrate how the collaboratively developed concepts of *being treated as “other”* and *being made inaudible* can advance our understanding of the forms, dynamics, and effects of racism in healthcare encounters. Because this paper focuses on the process of racialization, it helps illumine the mechanisms of subtle racism, which, as study results suggest, can damage healthcare users, cause a loss of trust in the system, and lead to invisibilization of racism in healthcare. By doing so, it draws attention to areas for change and transformation, to larger power structures that must be challenged in order to ensure responsive and equal healthcare for all healthcare users. The application of CBPR and, particularly, the engagement of racialized healthcare users in the research process offered pathways for analyzing the subtle, otherwise hard-to-detect mechanisms of racism, and for learning from the wisdom of situated knowledges.

## 1 Introduction

While an established body of literature, largely from the North American and Australian contexts, demonstrates how encountering racism in healthcare is burdensome (1), the impact of racism on healthcare interactions remains gravely understudied in Europe, particularly in Germany (2, 3). A limited number of studies from recent years (4) provide selective insights indicating that racism in the context of German healthcare can negatively affect the quality and accessibility of healthcare and the overall health of healthcare users. Notably absent from the existing literature and from the prevalent German scientific discourse at large are the perspectives of racialized communities, with the single exception of the Afrocensus, which is the first community-based survey of anti-Black racism in Germany and covers many fields, including healthcare (5). The study presented in this paper tends to fill this gap by examining the ways in which racism is experienced by healthcare users who are Black, African, Afro-diasporic, and/or (perceived or self-describing as) Muslim; it aims to advance our understanding of racism’s forms, dynamics, and effects. Accordingly, the study addresses the following research questions: In what ways and with what consequences do healthcare users experience racism? How do they encounter racism? In order to examine racism from the perspectives of healthcare users affected by racial injustice, the research design is situated within a Community-Based Participatory Research (CBPR) approach. CBPR seeks to democratize the production of knowledge by actively engaging the individuals whose experiences and health are under study in all phases of the research process.

The collaboratively developed analytical framework, which seeks to address dynamics of *being seen and treated as “other”* and *being made inaudible*, contributes to an empirically grounded conceptual understanding of healthcare users’ experiences of racism. The methodological approach presented in this paper therefore illumines the subtle mechanisms that lead to the (re)production of racial inequalities and the normalization (invisibilization) of racism in healthcare. Our study also responds to scholarship that critiques current research on racism in healthcare for its lack of theoretical focus on processes of racialization (6, 7).

This paper proceeds in the following manner: First, we provide a brief overview of racism in healthcare, followed by a short description of the German context. Second, we describe CBPR as a research approach and how it was implemented in our study. Third, we present the findings, which draw primarily on participatory focus group discussions with 14 healthcare users who are Black, African, Afro-diasporic, and/or (perceived or self-describing as) Muslim. In the final section, we locate the resulting concepts theoretically and empirically in the wider body of literature and offer some concluding thoughts.

## 2 Racism and healthcare

The discussion of racism in our study draws on an understanding of racism as an historically emergent and society-wide phenomenon that expresses relations of dominance. These power relations are supported by mechanisms of categorization, naturalization, binary opposition, and hierarchization, as well as by the attendant ideologies, and they are maintained by discourses and practices. As such, racism legitimizes, stabilizes, and reproduces material and symbolic exclusions (8–11). Racism in healthcare, as in other institutions, operates at multiple, interrelated levels, ranging from the individual to the structural within existing structures (7), and constitutes a major barrier against achieving equitable healthcare (1).

An established body of literature about racial differences in health outcomes has demonstrated how perceived racism can negatively affect life expectancy as well as physical and mental well-being (12–15). Complementing these findings, more recent research shows that individuals affected by racism may internalize racist values and biases, which in turn can seriously impact their own self-worth and lead to negative physical and mental health outcomes (4, 16).

Although subject to more scarce scholarly examination, racism in healthcare—the focus of our study—can occur in various forms, such as barriers to access, lack of diversity, poor uptake, and poor healthcare quality (17). A scoping review by Hamed et al. (1) found that the US, Canada, and Australia dominate the research conducted in this field and indicated that racism can occur in various healthcare contexts, such as hospitals and general practitioners (GPs), and in interactions between different actors; it also indicated that racism can negatively affect both racialized healthcare users and racialized healthcare staff (18–22). For instance, racialized healthcare users encounter an overall poorer quality of healthcare and are undersupplied when experiencing pain (21) or when in dental care (23). Furthermore, they experience racism in differentiated ways, which can range from being excluded from decision-making processes in healthcare interactions (24) to receiving a general lack of respect (25) and having one’s symptoms and complaints dismissed (6). Studies report that healthcare staff tend to homogenize racialized healthcare users by viewing them as irrational (26), problematic (especially in the case of Muslims) (27), and “too emotional” (28). The majority of the empirical work conducted in this field indicates that racism not only can contribute to the (re)production of health inequalities (29), but also can impact future patterns of health service use by influencing levels of trust and patient satisfaction (20, 30, 31).

The small number of studies of European countries, mainly located in the United Kingdom and Sweden, illustrate the problematic prevalence of racism in their healthcare systems (6, 32–35). According to the aforementioned review, current research on racism in healthcare generally lacks a theoretical focus on the processes of racialization, which makes it difficult to conceptualize racism and to understand how racial inequalities are (re)produced in healthcare encounters (1).

This remarkable lack of data on racism and healthcare in Europe may be explained by the fact that the usage of racial and ethnic categories in data production was rejected in European countries (except for the United Kingdom) following the Second World War, on the grounds that using these in official data is inherently racist (36). Both academic and public discourses preferred the rhetoric of migrant integration over deeper engagement with themes of “race” and racism (37, 38). The reluctance to work with “race” and racism as scientific categories attempts to position racism as a problem that belongs to the past, making current analysis of racism as an urgent contemporary problem far more difficult.

## 3 Racism in the German (healthcare) context

The German context is particularly challenging for researching racism. Temporal, social, and spatial externalizations have for a long time characterized public and institutional engagements with racism in Germany (39). Construed as peculiar to the National Socialist period (40) (p. 489), racism was frequently relegated to the past. Concomitantly, it underwent a form of social displacement onto right-wing extremism and is thereby construed as a problem identified with the periphery of society (39). Finally, the spatial displacement that occurs through the representation of racism as a problem of former colonial powers or of the US as a neocolonial entity has effectively erased German colonial history and its effects on contemporary racist discursive formations and practices (39). These developments produced a political climate within Germany that enhanced the denial and silencing of racism (40), a climate further evidenced in the fact that the German state has avoided the keeping of statistics on racial discrimination and introduced the category of “migration background” into micro-census statistics only in 2005 (41). Research at the intersection of health and migration therefore includes work concerned with the living conditions of migrants in Germany. However, such studies only marginally account for forms of racial discrimination as *eo ipso* explanatory variables for health disparities, and their focus lies more squarely on the specificity of subjects as migrants (42). Analytical perspectives that center too forcefully on the process of immigration itself risk a mode of interpretation that is overly culturalizing. Health disparities are then explained reductively in terms of “cultural” differences and the concomitant differences in “ways of life” (43). An explanatory approach of this sort risks promoting racist categorizations and remaining silent about existing structures, which should instead be problematized as risk factors in health (44). Moreover, racialized communities that include individuals who have not migrated, such as Black Germans and German Sinti, are not covered by research focusing migration.

Consequently, there is a paucity of empirical work on racism in German healthcare. A limited number of qualitative studies on the experiences of Black patients and patients with “migration backgrounds,” and on the perspectives that “white primary care physicians” bring to their interactions with these groups of patients, provide initial insights into racism in these settings (45–47). Hamed et al. (6) analyze experiences of racism among patients in Sweden, Germany, and Portugal as a form of structural violence that negatively influences access to healthcare as well as treatment. Other qualitative work focuses on barriers to treatment and care for asylum seekers and points to discrimination by calling attention to experiences of rejection, delays in treatment, medical malpractice, and language barriers, though this work does not explicitly mention racism (48, 49). Another study by Schödwell et al. (50) points to the economic and organizational structures of healthcare (a lack of time and staffing) that may contribute to the (re)production of racism in healthcare. Finally, there is a particularly pronounced lack of studies carried out *with*, rather than *on*, persons affected by racial injustice in German healthcare. A notable exception is the aforementioned Afrocensus, which demonstrates how othering processes operate as the basis for anti-Black racism in German healthcare, and how racist patterns of thinking and acting hinder access to healthcare on structural and individual levels (5).

The study presented in this paper addresses this gap by focusing on the experiences of healthcare users who are Black, African, Afro-diasporic, and/or (perceived or self-describing as) Muslim while actively engaging them in the research process. Since racism is considered a silent and silenced phenomenon in European healthcare (38), we hope to contribute to breaking that silence by advancing the conceptual understanding of its forms, dynamics, and effects and by drawing attention to areas in need of change and transformation. The results from this study are likely to be transferrable to other healthcare systems in Europe.

## 4 Materials and methods

### 4.1 Study design: community-based participatory research

Historically, the practice of Western academic research on racially marginalized communities has been coercive, deceptive, and occasionally harmful. Western academia has attempted to “justify” slavery, “prove” the racial superiority of white people, and has negated the humanity of individuals and communities of color (51, 52). In the German context, the medical experiments of Robert Koch in East Africa (53) constitute only one of numerous examples of unethical research practices. To this day, the colonial past affects research practices and is to be reckoned with in disciplines as diverse as sociology, medicine, and anthropology (54, 55). For example, material and discursive disadvantages are reinforced by the misunderstandings and misconceptions that are held by Western academic researchers (56). These misunderstandings and misconceptions are often expressed in scholarly narratives that produce othering effects and that transmit images of migrants as (potential) “carriers of disease” (57) or as “hard-to-reach” populations (58). Historical as well as current abuse in the practice and use of research has fostered mistrust within racially marginalized communities and magnified power imbalances; instead of being co-created and shared, research data have been extracted from communities (59). These harms have been described by Kristie Dotson as “epistemic oppression” (60). This term, which has been employed in order to theorize the ways in which specific population groups are suppressed with respect to their power to contribute to knowledge creation, adds a new domain of explanatory power to the rich tradition of postcolonial theory on epistemic violence (61).

It is in view of this background that our study applies CBPR. At its core, CBPR seeks to combine the expertise and knowledge of researchers and of those whose knowledge and subjectivity have historically been denied; it does this in the service of knowledge democracy and lasting social change (62, 63). CBPR draws inspiration from the contributions of critical methodologies in order to propose a set of principles that can be applied to address epistemic injustice in research: it starts from community priorities and builds on community strengths; it promotes a power-sharing process, fosters co-learning and capacity-building, and embraces cultural humility (64, 65).

In line with CBPR, our study involves the perspective of racially marginalized communities as “subaltern standpoints” (66) in defining the research questions, in data collection and analysis, and in interpreting and disseminating the research findings. Thus, it applies a mixed methods approach in using both inductive and deductive theory to guide the overall research process.

#### 4.1.1 Recruitment: peer researchers and participants

Following CBPR, it was crucial to have on the research team healthcare users who are affected by racism. The core team that was formed consisted of one researcher, who was a representative of the research institution, and two peer researchers (67); these are the three authors of this paper. The peer researchers were recruited from migrant organizations. The following criteria were considered in their selection: the persons are affected by anti-Black and/or anti-Muslim racism; they are well connected in their respected communities; and they speak German, English, and either French or Arabic. The peer researchers were trained by the representative of the research institution in research ethics and data protection, research methods and focus group facilitation techniques, and data management and data analysis. They were employed on an hourly wage basis for the entire duration of the project. We used the training sessions to collectively think through and critically discuss the original study design. In this way, the research questions that had been formulated in the project proposal were further elaborated and differentiated by the peer researchers and were coordinated with the cooperating migrant organizations. This step was crucial for ensuring that the research questions are relevant to the research community, as the original project proposal had only been written due to a gap in the scientific literature.

The recruitment of 14 study participants, who are Black, African, Afro-diasporic, and/or (perceived or self-describing as) Muslim, was supported by the existing networks of the two peer researchers. A maximum diversity sample was employed, meaning that study participants were chosen to include the greatest possible range in terms of age, gender, education, language, residency status, length of residency, and health concerns. This method ensured that a rich range of experiences was obtained.

#### 4.1.2 Advisory board

To ensure the methodological quality of the participatory assessments and evaluations, an advisory board was convened. It consisted of three independent scholars (two of whom were scholars of color) who specialized in migration, racism, and participatory health research. Throughout the entire research process, the research core team was supported by the advisory board members, who co-designed the peer researchers’ training and the focus groups, and provided feedback on the research questions, preliminary findings, and the research dissemination plan.

#### 4.1.3 Data collection and analysis

Data collection comprised a total of six focus group discussions (68) and a total of 14 participants: these participants were organized into two subsamples of seven participants each (subsample one: Black, African, Afro-diasporic participants; subsample two: participants perceived or self-describing as Muslim), and there were three focus group discussions for each subsample. Following the CBPR principles of participation that is as expansive as possible and research that is empirically anchored—and thus proximate to the experiences of the study participants—the focus groups were conducted in two languages (German and English) and developed by us (as core team) sequentially, in iterative cycles of action and reflection (59, 69). Each session was built on the previous one and conducted with the purpose of examining general barriers to accessing healthcare. Questions concerning barriers, facilitators, experiences of discrimination and their effects, and coping strategies were discussed. Participant experiences of racism were shared voluntarily. Focus group meetings lasted 90 min each and were conducted as video conferences, due to the COVID-19 pandemic, between April and July 2022. Their facilitation was dialogically shared between us (researcher and peer researchers), as suggested by Krueger & Casey (70). Having a co-researcher as a co-facilitator in each subsample served to encourage discussion and build trust. Peer support of participants with shared experiences was also an enabling factor for discussion of difficult issues such as racism. In both subsamples, mutual engagement became more intense over time—participants seemed to contribute longer and more varied accounts in the second and third focus group discussions. Participants supported each other by agreeing with each other by name, by sharing similar experiences, and by building on each other’s comments—verbally when painful experiences were shared and non-verbally through active listening and nodding. In the collective process of discussing, listening, and learning from each other, they raised critical questions, identified their individual experiences as shared, and engaged in collective sense-making. Some participants described the focus group discussions as empowering. The audio recordings of the focus group discussions were transcribed and pseudonymized, and participants had the option to choose their own pseudonyms. The quoted passages in this article that we (the authors) translated from German are indicated by asterisks (*).

In line with CBPR, we (as a core team) wanted to work against research practices that only position the communities we worked with as marginal. We were interested in learning from each other and from the study participants. Thus, a collaborative approach to qualitative data analysis was applied by adopting the DEPICT model (71). The title acronym (DEPICT) is an active verb that means “to describe using words,” which is a core activity within data analysis. DEPICT has six sequential steps: Dynamic reading, Engaged codebook development, Participatory coding, Inclusive reviewing and summarizing of categories, Collaborative analyzing, and Translating. DEPICT is designed to enhance methodological and ethical rigor by involving stakeholders with varying levels of research proficiency in data analysis. We analyzed the focus group material in accordance with the DEPICT concept, in which inductive and deductive steps complemented one another. This means, e.g., that the transcripts were coded inductively and the elaborated categories were conceptually diversified by applying postcolonial theoretical frameworks, namely those of othering and silencing. Feedback loops with the focus group participants, as well as with the advisory board, were integrated in the process of analyzing in order to further diversify the epistemic standpoints from which data was interpreted (72), and confront othering in data interpretation and data representation (73). The data analysis was supported by using the MAXQDA 2022 analysis software (VERBI Software 2021).

### 4.2 Ethical considerations, reflexivity

Ethical approval was obtained from the Ethical Review Committee of the DeZIM-Institute (approval no.: DI-2022-0003). All participants received verbal and written information on the study and on the way in which their statements in the focus groups would be treated. All participants gave informed consent in verbal and written form and received explanations of how we would safeguard their confidentiality and anonymity. They were also informed about the voluntary nature of their participation. Given the sensitivity of their discussions of experiences with racism, participants were provided with contact details for consulting centers where they could contact a therapist or psychologist if they wished. They were additionally given the contact details of the researcher and peer researchers in case they had additional questions or inquiries. Spaces for reflexivity and peer researcher support, in the form of feedback loops within the core team, with the advisory board, and with external supervision, were available throughout the research project.

## 5 Results

In the collaborative data analysis, two concepts emerge as central: *being seen and treated as “other”* and *being made inaudible*. Both concepts, along with their respective sub-items, are presented below and, in the concluding discussion, are located empirically and theoretically in the wider body of literature.

### 5.1 Being seen and treated as “other” in healthcare encounters

They just don’t see that we’re individual human beings, that every person is different. [...] Just because that one patient [...] is resistant to pain doesn’t mean that all people from Africa feel no pain. (Paul)*

Paul, a young Black male focus group participant born in Germany, illustrates his experiences with medical professionals by highlighting how Black people are de-individualized, homogenized as a group, and construed as inherently different, as “others”—in this case, as insensitive to pain. The same topic is discussed by Lilliane, a young Black female participant who, like Paul, was socialized in Germany. She addresses the alienation that is imposed on Black people when she refers to the way that healthcare providers locate Black people in economically poor countries of origin with no effective healthcare systems. This results, in her experience, in their assumption that Black people are used to dealing with pain and therefore have a higher pain tolerance and can be left waiting longer for treatment—as she was.

While Black study participants reported being attributed insensitivity to pain, study participants perceived or self-describing as Muslim shared experiences of encountering medical staff who presumed that they are oversensitive to pain. Pari, a female participant, reported a case where a male family member accompanied by his daughter (who was wearing a hijab) was denied treatment by the GP and sent home. Pari assumes that his complaints were not taken seriously. This had grave consequences:

He [the GP] said: “No, no, don’t act like that.” [...] And at some point he [her family member] could no longer breathe. This time they had to take him to the hospital in an ambulance. Then it emerged that he really did have Covid. [...]. He had to be ventilated immediately. [...] He was triple vaccinated. [...] They [the hospital emergency department] even called the GP to ask whether it’s really true or whether it’s just faked. Eventually the GP exclaimed: “Oh yes, the PCR test was positive!” [They responded:] “Thank you. We don’t need this information. He’s already dying.” (Pari)*

In contrast to such a denial of treatment, Rose, a Black refugee woman in her 40s, describes how she was offered an HIV test at the gynecologist without having asked for it. As HIV testing is not offered to women on a regular basis in German healthcare, she assumes a connection to her blackness and to prevailing images of Black women as hypersexual and carriers of diseases, as she added later in the same focus group. The accounts presented by study participants show how different markers, such as being perceived as Black or wearing a headscarf, can lead to different assumptions in healthcare interactions amidst similar circumstances: while Black female participants report being hypersexualized by medical professionals, female participants perceived or self-identifying as Muslim describe how they are being denied an autonomous sexuality. Taslima, a female Muslim participant who is in her 40s and wears a headscarf, illustrates how a gynecologist automatically classed her into a group of “women from a certain culture.” Based on the assumption that, comparatively speaking, she is not sexually active, the gynecologist concluded that there was no reason to provide her the healthcare service she had asked for:

One time, when I asked the gynecologist for a STI [sexually transmitted infection] test, she was stunned and said I shouldn’t worry too much as that’s rather unlikely for women from my culture. Women from my culture? (Taslima)*

Additionally, the discursive narratives about Muslims that enter interpersonal communication in healthcare settings can indicate gender-specific influences. Ahmad, a queer focus group participant (pronoun they), described how they were read as Muslim and associated with Islamist attackers, and how this negatively impacted the quality of their medical treatment.

*He prescribed a medication that has some side effects, like mental health side effects. “You need to be careful because one of the people in the plane of 9/11 was taking this medication.” He was making the association between me and the terrorist instead of saying people can get angry, get depressed, etc., which is written on the Beipackzettel [package slip]. […] This assumption about me resulted in not answering my questions, not giving me the best treatment and the general feeling of not being cared for. (Ahmad)*

Study participants recognize in these experiences a repeating pattern. “They’re not isolated cases” were the words used by Paul to express his observation that everyone engaged in the focus groups has a story to tell and has also experienced something of this sort several times. Paul also described how, regardless of his own appearance and conduct, it is not always possible to escape or to control or influence processes of being treated differently, as “other.”

### 5.2 Being legally treated as “other” in healthcare

Several study participants who are seeking asylum described a specific mode by which the act of marking and treating someone as different, as “other,” is normalized and institutionalized—mainly by the German healthcare related legislation:

*There are also structural factors, like the Asylum-Seekers’ Benefits Act, which are already established and block the access to medication or medical treatment. The person who doesn’t have “this and this” is not allowed to have access to “this and this.” These laws help the authorities like Ausländerbehörde [Immigration Office] and Sozialamt [Social Security Office] to say, “We are acting according to the law. It is in the law. So, we are not acting discriminatory.” (Rose)*

Rose refers in this quote to the Asylum-Seekers’ Benefits Act (Asylbewerberleistungsgesetz, AsylbLG), which regulates access to healthcare for asylum seekers in the first 18 months of their asylum-seeking procedure and limits healthcare to acute care, pain treatment, pregnancy care, and vaccinations. Rose sees in this regulation a legitimization for the denial of equal healthcare.

Some participants described how the legally prescribed dispensation of physicians’ certificates of medical treatment (Behandlungsscheine) by the Social Security Office (Sozialamt)—legal practice in seven federal German states—leads to delays of their medical treatment. This was articulated by Aziz, a Muslim participant:

*These are horrible moments because I was really sick and I needed my medication. At the end I got the Schein [certificate] but too late, after a week. (Aziz)*

Other participants spoke of an additional effect of this legal practice by highlighting how their treatment thus depends on the decisions of medically unqualified personnel. Peroz, a middle-aged man who is seeking asylum and is perceived as Muslim, stressed how he had to convince a case worker (Sachbearbeiter) at the Social Security Office (Sozialamt) again and again that he really does need to see a doctor.

Even when Social Security Office (Sozialamt) clerks are persuaded to grant a treatment certificate (Behandlungsschein), there is no guaranteed access to the required medical treatment—even in cases of goodwill on the part of the doctor. This was highlighted by Taslima:

However, he [the dentist] said that he’s not allowed to treat my teeth because I’m still in the asylum procedure [Asylverfahren], and treatment costs are not covered. He is only allowed to remove my teeth. (Taslima)*

These accounts exemplify the various ways in which AsylbLG operates: through institutions such as the Social Security Office; through GP surgeons and their staff; through the refusal to approve or through the reduction of medical services; and through improper ex ante assessments.

### 5.3 Internalizing the experiences of being seen and treated as “other”

I called them […] we were arranging an appointment […] until they asked me for my name [...]. Then they said: “Well, the appointment in question is not possible. Please call back at a later date.” That was just a bit painful—on the one hand because it means I have to wait longer for an appointment of course, on the other because my name is part of my identity [...]. It grates on my dignity, to be honest. (Hamid)*

Focus group participants often described the painfulness of their individual, persistent experiences of being seen and treated as different, as the “other.” The above accounts of Hamid, a young Muslim man socialized in Germany, show how these experiences negatively affect his perceived sense of (human) dignity and self-worth. Some participants highlighted how these experiences can trigger a sense of inferiority and self-doubt, a view of their own self as “other”:

*I am asking myself why, and of course this is one of the mental health impacts of discrimination, being sick in healthcare and not feeling cared for. So, that’s why you start questioning yourself. Why is this happening? Is something wrong with me? Did I do something wrong or should I do more to present myself in a way that the doctors care about me? (Ahmad)*

Ahmad spoke of how these experiences made them question themselves and locate the issue within themselves. They were asking themself if enacting a more assimilated appearance would lead to receiving less-degrading treatment. Paul reported how the experiences of being treated differently made him think that he is not in fact “worthy” of good treatment:

Like I said, you are not respected and at some point you don’t respect yourself anymore either. You end up saying, “Woah, there’s no point going to the doctor’s, I’d rather stay home and endure the pain.” (Paul)*

This quote demonstrates most forcefully how Paul avoided seeking treatment in order to prevent mistreatment. The following section further examines the choice to not utilize the healthcare structures.

In conclusion, the described dynamic of being seen and treated as “other” constitutes a multi-step process, which renders study participants as “others,” categorizes them as deviant and not belonging, and consequently devalues them. This can cause ongoing harm: it can lead to the dismissal of symptoms and complaints, to poorer quality of healthcare, or to delays in treatment.

### 5.4 Being made inaudible

And so you very often have a situation like I experienced at the doctor’s, where they ask you whether you can read these documents yourself. They’re having a conversation with you in German, but they have already presumed illiteracy [...] because I have a headscarf. Or they absolutely want to make decisions about my body. (Taslima)*

In this quote, Taslima describes how her voice was ignored by the doctor in a direct interpersonal encounter. The reason for this, she states, is that she was wearing a headscarf, which in her view serves as a marker for a culturalized imagination of Muslim women’s educational deficits. She was not given the option to express her needs on equal terms. The doctor made the final decision for her. This dynamic is described by multiple participants, who spoke of not being seen as agentive subjects, not being taken seriously with their concerns, and not being listened to by medical professionals.

Even if our focus group participants do not literally remain silent, the knowledge they articulate is perceived to be devalued and actively ignored:

He [the doctor] said: “I want to do a vaginal sonography.” [...] “I don’t want vaginal sonography.” Then he said to me: “How long have you been in Germany? Well well, still not integrated.” [...] “No, I don’t want you as a man examining me vaginally. I trained in sonography myself and know that there is another way.” He said, “If you don’t like a vaginal examination, I can’t do anything for you. Bye.” [...] I knew that bleeding is not a good sign, and I also had pain. After a week, the baby was gone. (Mahnusch)*

The accounts of Mahnusch highlight how her requests were disregarded and not heard by the doctor. Instead, her experiences and knowledge are devalued by him. As a result, she did not receive adequate healthcare treatment, which in her case has led to serious health consequences.

Some study participants emphasized the importance of how they are spoken to in healthcare encounters.

I have observed how the doctor relates to other clients in the office. And then how he relates to me or other refugees I have accompanied. […] The tone changes. […] It becomes very loud, and then, “Can you understand?” (Peroz)*

Peroz reported a case illustrating how—in his eyes—power relations become evident through tonality and speech, which covertly transmitted an othering message. He was perceived as someone who is less knowledgeable, and this automatically resulted in the presumption that he is incapable of understanding. Similarly, other participants described being spoken to in a rude or condescending manner, as well as being confronted with dismissive looks by medical professionals, which was said to be intimidating. These experiences were perceived as an enforcement of division, as solidifying a hierarchy of those who are knowledgeable and agentive and those who are unknowledgeable and encouraged to limit their speech.

As a reaction to the recurring experiencing of being treated differently or of not being heard, healthcare users involved in the study reported how they began to refrain from articulating their concerns. Some remained silent because they assumed that their counterpart would not have an appropriate understanding of their concerns. This was highlighted by Ruth, a Black migrant woman in her 60s:

I try to ignore these experiences and to move on. [I say to myself:] What else do you want to do? Here you are nobody. Who will listen to you? (Ruth)*

Others remained silent in order to prevent adverse consequences.

*Sometimes you want to complain but you don’t want to put the word racism […] you still have the burden of being discriminated but still try to put it in a nice way because you are still a patient. You still want to receive the service […] especially if you are in the middle of a treatment that started long ago […] you don’t want to interrupt this connection. […] You are depending on the doctor, especially when you have a chronic disease […] it becomes very scary to start all over again. (Ahmad)*

In this quote, Ahmad articulated their inhibition in naming racism out of fear of jeopardizing the doctor-patient relationship. The power of the doctor, which lies in his expert knowledge and in the vulnerability of the participant, who suffers from a serious chronic disease, creates a dependency that makes it especially difficult for the participant to resist disparagement or disrespect from a professional.

Participants with legal considerations, such as an asylum application, described particularly difficult circumstances.

When you are in such a dependent situation, like at the doctor’s or at the [Social Security] Office [im Amt], you’ll think eight times whether you really want to speak up against it, because you might not be able to choose a different doctor due to your asylum process [Asylverfahren]. (Taslima)*

Taslima recounted the structural dependencies that exist across situations and that shape interactions in healthcare settings: as an asylum seeker, she and her healthcare depend not only on doctors but also on the AsylbLG, in conjunction with the Social Security Office (Sozialamt) as the institution that decides on the dispensation of treatment certificates. She describes how this impacts her behavior and how she has to weigh between bringing up her experiences of racism and, in anticipation of negative consequences, not naming these same experiences.

Other study participants affected by incidents of ignorance and disrespect in their healthcare encounters reported how these repeated experiences make them lose trust in the medical system and influence their capacity to act. They abstain from existing treatment options despite their needs. This was expressed by Lilliane:

Why should I go to the doctor? Why should I have trust anyway? [...] When I have already had negative experiences with a white doctor or a white institution, why should I trust that they suddenly want something good for me? (Lilliane)*

This quotation illustrates how a dual mechanism of exclusion operates: first, healthcare users are discriminated against in encounters with medical professionals; second, they avoid making use of healthcare structures, and thus do not receive treatment, due to prior experiences in which they were treated as “other” or ignored, not heard.

In conclusion, the described dynamic is reconstructed in two forms: (1) study participants do not feel seen as knowing subjects and therefore consider themselves not listened to, not heard by medical professionals; (2) study participants report refraining from articulating their concerns based on prior negative experiences or fear of adverse consequences.

## 6 Discussion

This article explores the accounts of 14 healthcare users who are Black, African, Afro-diasporic, and/or (perceived or self-describing as) Muslim regarding their experiences of racism in healthcare encounters. It aims at understanding the forms and dynamics that lead to the (re)production of racial inequalities in healthcare-related contexts. In the data analysis, two dynamics emerge as central: *being seen and treated as “other”* and *being made inaudible*. In the following discussion, these dynamics are theoretically and empirically located in the wider body of literature. Finally, some concluding thoughts are offered.

The study participants’ experiences of *being seen and treated as essentially “other”* in healthcare interactions can be situated in postcolonial theories that analyze othering, such as those of Said (74) and Spivak (61, 66). Othering describes a process of differentiation by which an imagined “we” is discursively produced in contradistinction to the “other” and the “alien.” This process, which posits the “other” as a deviation from a normative “we” and places the “other” in a relationship of hierarchy (74), is central to the operation of racism, which depends on the differentiation of subjects into imagined groups based on social constructions of race, culture, nation, or religion (10, 75). The data illustrate how medical professionals use so-called markers, such as name, skin color, and real or surmised religious affiliation, to infer a particular group membership and make stereotypical attributions that de-individualize and homogenize the individuals who participated in the study. Study participants are thus construed as essentially different, as not belonging and alien (fremd), which is to say, as “other.” The data indicate how they are viewed as illiterate, as sexually oppressed, as Islamists, as hypersexual and carriers of diseases, and as oversensitive or insensitive to pain. These results are also reflected in healthcare-specific studies in different contexts: a UK study (33) of the experiences of Pakistani healthcare users demonstrates how they feel homogenized and reduced to the category “Muslim” by nurses; in a US study, Black healthcare users report feeling stereotyped as less intelligent (76); in a study focusing on racism within German healthcare, Black patients report having hypersexuality and insensitivity to pain attributed to them by healthcare staff (5).

Postcolonial theoretical formulations make connections between the attributions that are processed in the data and the racist discursive conditions of knowledge: these formulations speak about how colonialism produced stereotypical images of “hypersexualized” and “pain-insensitive” Black bodies (77), or of the headscarf as a symbol of the oppression of women (78). Other discourses are shaped by the contemporary and have already been broken down in other German studies, such as the discourse that frames migrants as “carriers of disease” (57). However, as the study results show, colonial and current narratives about Black people, Muslims, and “cultures” can also connect intersectionally, as with the image of a “sexually oppressed Muslim woman” as described by Taslima or of a “dangerous Muslim man” as described by Ahmad.

Some of the accounts presented by study participants indicate how this negative stereotyping can influence the ability of medical professionals to see and treat them as knowledgeable subjects, and how it can lead to the dismissal of their symptoms and complaints, to misdiagnoses, and to poorer quality of healthcare or delays in treatment, as has already been documented in other studies (5, 6, 21, 23, 45, 79). Our study expands previous empirical research concerning the specifics of anti-Muslim racism, in which it is shown how othering is expressed differently in relation to Black healthcare users and healthcare users perceived or self-describing as Muslim, and how it can lead to different forms of racist discrimination in healthcare. The data suggest that Black female study participants are often hypersexualized, while the autonomous sexuality of female study participants who are perceived as Muslim is denied. As a result, Black female study participants are often offered HIV tests. In contrast, medical professionals see no reason to carry out tests for sexually transmitted infections (STIs) on Muslim women who participated in the study.

Study results suggest that othering is experienced at different, interlocking levels, such as the interpersonal, the institutional and the structural. These mutually reinforcing intersections are best exemplified in our study by accounts of participants who are asylum seekers. Data draw attention to the micro-level effects of asylum policies by illustrating how these policies act through interpersonal encounters and manifest in various forms. The data show how the legally required issuing of treatment certificates by social welfare offices leads to delays in, or even to prevention of, medical treatment for sick people, and how staff who are not medically qualified actually decide whether medical treatment is necessary. In addition, the restrictions on health services for asylum seekers that are enshrined in the Asylum Seekers Benefits Act (AsylbLG) apply. This means that certain health-related services are not covered for some study participants. These results are consistent with a limited number of qualitative studies that point to discriminatory effects of the AsylbLG (48, 49, 80), though the last two do not explicitly mention racism.

Data show that recurring experiences of being othered can trigger a sense of inferiority and self-doubt (6) and a view of one’s own self as “other,” as described by our study participants Paul, Hamid, and Ahmad. This is an example of how racism remains effective within dominated subjects as well (81) and how othering is internalized. This can seriously impact self-worth and has been linked to negative physical and mental health outcomes in recent studies (16, 82). Thus, our study results draw attention to the necessity of examining internalized racism, as it is paid relatively limited attention in healthcare research until now (83).

General consequences of othering practices, as described by participants in our study, are a loss of trust in the healthcare system and a deterioration of health. This resonates with studies showing how perceptions of unequal treatment on racial grounds can discourage healthcare users from accessing healthcare services: they lose trust in the healthcare system (5, 6, 20, 84), which in turn leads to the likelihood of avoiding or delaying healthcare-seeking, even when healthcare is needed (85–87), and to the deterioration of mental and physical health (5).

Spivak’s (61) seminal intervention, “Can the Subaltern Speak?,” helps to connect the above-discussed result with the second analyzed concept: *being made inaudible*. Spivak demonstrates how individuals or groups who are construed as “others” confront serious disadvantages. As a result of the epistemic violence (61) that accompanies othering, those constructed as “others” are not recognized or heard in their positions as knowers; they are instead subjected to silencing and/or discrimination. Kien Nghi Ha et al. (88) point out how “the white norm ([d]ie Weiße Norm) speaks, judges, and remains invisible within the powerful process of othering,” and “how ‘others’ are spoken about, analyzed, and devalued and thus become supposedly silent, faceless objects” (88) (10, transl.). Building on Spivak’s (61) use of the expression “epistemic violence,” Dotson (89) develops an account of epistemic violence by analyzing different ways in which silencing operates at a micro-level. Following the analysis of Dotson (89), two different kinds of silencing can be identified in our data: study participants—owing to negative prejudices and stereotypes—are routinely not recognized as knowing subjects and have their agency denied by medical professionals (testimonial quieting); study participants refrain from articulating their concerns due to the apparent ignorance of their interlocutor, or to a fear of adverse consequences (testimonial smothering, or coerced self-silencing). As stated by Dotson (89), silencing dynamics can be identified by paying attention to racial micro-aggressions, which serve as subtle forms of epistemic violence because they implicitly encourage people to limit their speech. Micro-aggressions, both in theory (90) and in our data, express themselves non-verbally (dismissive looks) as well as through speech (questions and comments conveying difference and subordination) and tonality (speaking loudly) and are experienced to be intimidating. According to Gerlach et al. (45) and Velez et al. (25), communicative dynamics can be reinforced on non-verbal levels such as “unfriendliness” or “a lack of respect.” As background, Aikins et al. (5) expect that the participants are not recognized as responsible speakers. Possible consequences of these processes are, as our data also show, that healthcare users are left out of decision-making processes (24) and that their symptoms and complaints are not taken seriously or are ignored by healthcare staff (6, 79).

As Dotson (89) argues, testimonial quieting and testimonial smothering can be understood primarily through analysis of power relations and other contextual factors that make silencing harmful in particular circumstances. In the context of healthcare, asymmetrical power relations become particularly clear in the silencing mechanisms described by study participants: healthcare settings are most frequently sought when people are sick and forced to seek help. Moreover, medical staff command medical resources and professional knowledge that situates them in a position of power over those seeking help (38). The dependencies and vulnerabilities that emerge from this situation make it particularly difficult for study participants—as evident in the accounts of Ahmad—to defend themselves against racist discrimination. This especially applies when there are additional legal dependencies, as is the case for study participants, such as Taslima, who are seeking asylum. Empirical research in healthcare is only now gathering a wealth of evidence of the multiple and far-reaching harms that silencing—a powerful dynamic that involves structural and individual elements, and that leads to the invisibilization of racism in healthcare—can cause to individuals and even entire groups. For example, Hamed et al. (6) analyze experiences of racism, as a form of structural violence, among healthcare users in Sweden, Germany, and Portugal. They demonstrate how two interrelated processes, namely unequal modes of access to resources and inequalities in power, can lead to the silencing of suffering. Qualitative studies with healthcare staff in Sweden and Canada have similarly documented a failure to bring up experiences of racism in the workplace out of a fear of adverse consequences (19, 32). Healthcare staff affected by racism instead suppress their emotions and feelings and acquiesce to the power structure in which they operate (19, 32). The added value of our study lies in having taken additional and more differentiated steps in documenting the relevance of this dynamic for the German healthcare context and extending the focus to healthcare users affected by racism.

### 6.1 Conclusion

According to Nazroo et al. (7) and Hamed and Bradby (91), current research on racism in healthcare lacks a theoretical focus on the processes of racialization, which makes it difficult to conceptualize racism and to understand how racial inequalities are (re)produced in healthcare encounters. The collaboratively developed analytical framework, which seeks to address the dynamics of *being seen and treated as “other”* and *being made inaudible*, contributes to an empirically grounded conceptual understanding of experiences of racism that have been reported by Black and (perceived or self-identified) Muslim healthcare users. Thus, it allows the reader to consider how racism is enacted through subtle mechanisms, which eventually (re)produce major barriers against achieving equitable healthcare and lead to the invisibilization (normalization) of racism in the field. By doing so, it draws attention to areas in need of change and transformation, to the importance of anti-racist policies that move beyond cultural and diversity competence approaches and that address racism at interpersonal, institutional, and structural levels, as these levels connect in mutually reinforcing intersections.

The application of CBPR, an approach to research that “is about who has the right to speak, to analyze and to act” (92) (p. 22), offered pathways to undo the silence, and to learn from the wisdom of situated knowledges, by engaging silenced voices in the research process (93). It helped to build relationships and trust and enabled a dialogical creation of knowledge. Due to the interlocking of perspectives, data could be collected in a context- and diversity-sensitive manner and interpreted from different perspectives. The qualitative study design also allowed extended views of the racialization processes or of latent, otherwise hard-to-detect dynamics, such as othering and silencing and their intersectional manifestations. The co-creation of knowledge is an empowering process that reaffirms study participants’ human status and, importantly, their human right to participate in research affecting their lives and health.

### 6.2 Limitations

This study design is consciously qualitative; it cannot and should not make any statements beyond the experience of participants. An additional limitation arises out of the conscious decision to work methodically with a data analysis procedure designed for as much participation as possible: the study certainly gained in quality through the interlocking of perspectives; however, DEPICT ends at a point where connections between the dynamics are not worked out. This is a fertile field for further exploration. The choice of peer researchers was also a crucial step that influenced the further course of the study: positively as well as negatively. Certain communities were thereby reached, while others were not.

## Data Availability

The datasets presented in this article are not readily available because of legal, ethical, and privacy restrictions. Requests to access the datasets should be directed to Tanja Gangarova, gangarova@dezim-institut.de.

## References

[1] Hamed S Bradby H Ahlberg BM Thapar-Björkert S. Racism in healthcare: a scoping review. BMC Public Health. (2022) 22:988. doi: 10.1186/s12889-022-13122-y, PMID: 35578322 PMC9112453

[2] Braun G Zeeb H. Gesundheitliche Dimensionen von Rassismus und Diskriminierung In: Spallek J Zeeb H, editors. Handbuch Migration und Gesundheit. Göttingen: Hogrefe (2021). 389–96.

[3] Namer Y Wandschneider L Stieglitz S Starke D Razum O. Racism in public health services: a research agenda. Front Public Health. (2022) 10:1039963. doi: 10.3389/fpubh.2022.1039963, PMID: 36504940 PMC9729794

[4] Deutsches Zentrum für Integrations- und Migrationsforschung (DeZIM) (2023). Rassismus und seine Symptome. Bericht des Nationalen Diskriminierungs- und Rassismusmonitors.

[5] Aikins M. Bremberger T. Aikins J. Gyamerah D. Yıldırım-Caliman D. (2021). Afrozensus 2020. Perspektiven, Anti-Schwarze Rassismuserfahrungen und Engagement Schwarzer, afrikanischer und afrodiasporischer Menschen in Deutschland. Each One Teach One (EOTO) e.V.; Citizens For Europe (CFE).

[6] Hamed S Thapar-Björkert S Bradby H Ahlberg BM. Racism in European health care: structural violence and beyond. Qual Health Res. (2020) 30:1662–73. doi: 10.1177/1049732320931430, PMID: 32546076 PMC7410275

[7] Nazroo JY Bhui KS Rhodes J. Where next for understanding race/ethnic inequalities in severe mental illness? Structural, interpersonal and institutional racism. Sociol Health Illn. (2020) 42:262–76. doi: 10.1111/1467-9566.13001, PMID: 31562655 PMC7028120

[8] Essed P. Everyday racism: a new approach to the study of racism In: Goldberg DT Essed P, editors. Race critical theories: Text and context. Hoboken, NJ: Blackwell (2002). 176–93.

[9] Hall S. Ideologie, Kultur, Rassismus. Ausgewählte Schriften 1. Hamburg: Argument (1989).

[10] Miles R. Rassismus. Einführung in die Geschichte und Theorie eines Begriffs. Hamburg: Argument (1991).

[11] Rommelspacher B. Dominanzkultur. Texte zu Fremdheit und Macht. Frankfurt: Campus (1995).

[12] Krieger N. Discrimination and health inequities. Int J Health Services: Plan Admin, Evaluation. (2014) 44:643–710. doi: 10.2190/HS.44.4.b, PMID: 25626224

[13] Paradies Y Ben J Denson N Elias A Priest N Pieterse A. Racism as a determinant of health: a systematic review and meta-analysis. PLoS One. (2015) 10:e0138511. doi: 10.1371/journal.pone.0138511, PMID: 26398658 PMC4580597

[14] Priest N Perry R Ferdinand A Paradies Y Kelaher M. Experiences of racism, racial/ethnic attitudes, motivated fairness and mental health outcomes among primary and secondary school students. J Youth Adolesc. (2014) 43:1672–87. doi: 10.1007/s10964-014-0140-9, PMID: 24903675

[15] Williams DR Mohammed SA. Discrimination and racial disparities in health: evidence and needed research. J Behav Med. (2009) 32:20–47. doi: 10.1007/s10865-008-9185-0, PMID: 19030981 PMC2821669

[16] James D. An initial framework for the study of internalized racism and health: internalized racism as a racism-induced identity threat response. Social and personality psychology. Compass. (2022) 16, 1–18. doi: 10.1111/spc3.12712, PMID: 39696797

[17] Elias A Paradies Y. The costs of institutional racism and its ethical implications for healthcare. J Bioethical Inquiry. (2021) 18:45–58. doi: 10.1007/s11673-020-10073-0, PMID: 33387263 PMC7778398

[18] Bastos JL Harnois CE Paradies YC. Health care barriers, racism, and intersectionality in Australia. Soc Sci Med. (2018) 199:209–18. doi: 10.1016/j.socscimed.2017.05.010, PMID: 28501223

[19] Beagan BL Bizzeth SR Etowa J. Interpersonal, institutional, and structural racism in Canadian nursing: a culture of silence. Can J Nurs Res. (2023) 55:195–205. doi: 10.1177/08445621221110140, PMID: 35746848 PMC10061608

[20] Ben J Cormack D Harris R Paradies Y. Racism and health service utilisation: a systematic review and meta-analysis. PLoS One. (2017) 12:e0189900. doi: 10.1371/journal.pone.0189900, PMID: 29253855 PMC5734775

[21] Mahabir DF O’Campo P Lofters A Shankardass K Salmon C Muntaner C. Classism and everyday racism as experienced by racialized health care users: a concept mapping study. Int J Health Services: Plan Admin, Evaluation. (2021) 51:350–63. doi: 10.1177/00207314211014782, PMID: 33949220 PMC8204040

[22] Mateo CM Williams DR. Addressing bias and reducing discrimination: the professional responsibility of health care providers. Academic medicine. J Association of American Medical Colleges. (2020) 95:S5–S10. doi: 10.1097/ACM.0000000000003683, PMID: 32889919

[23] Sim W Lim WH Ng CH Chin YH Yaow CYL Cheong CWZ. The perspectives of health professionals and patients on racism in healthcare: a qualitative systematic review. PLoS One. (2021) 16:e0255936. doi: 10.1371/journal.pone.0255936, PMID: 34464395 PMC8407537

[24] Peek ME Odoms-Young A Quinn MT Gorawara-Bhat R Wilson SC Chin MH. Race and shared decision-making: perspectives of African-Americans with diabetes. Soc Sci Med. (2010) 71:1–9. doi: 10.1016/j.socscimed.2010.03.014, PMID: 20409625 PMC2885527

[25] Velez D Palomo-Zerfas A Nunez-Alvarez A Ayala GX Finlayson TL. Facilitators and barriers to dental care among mexican migrant women and their families in North San Diego County. J Immigr Minor Health. (2017) 19:1216–26. doi: 10.1007/s10903-016-0467-2, PMID: 27456045

[26] Vydelingum V. Nurses’ experiences of caring for south Asian minority ethnic patients in a general hospital in England. Nurs Inq. (2006) 13:23–32. doi: 10.1111/j.1440-1800.2006.00304.x, PMID: 16494664

[27] Morel S. Inequality and discrimination in access to urgent care in France: ethnographies of three healthcare structures and their audiences. Soc Sci Med. (2019) 232:25–32. doi: 10.1016/j.socscimed.2019.04.028, PMID: 31048193

[28] Lyons SM O’Keeffe FM Clarke AT Staines A. Cultural diversity in the Dublin maternity services: the experiences of maternity service providers when caring for ethnic minority women. Ethn Health. (2008) 13:261–76. doi: 10.1080/13557850801903020, PMID: 18568976

[29] van Ryn M Burgess DJ Dovidio JF Phelan SM Saha S Malat J. The impact of racism on clinical cognition, behavior, and clinical decision making. Du Bois Rev. (2011) 8:199–218. doi: 10.1017/S1742058X11000191, PMID: 24761152 PMC3993983

[30] Freeman R Gwadz MV Silverman E Kutnick A Leonard NR Ritchie AS. Critical race theory as a tool for understanding poor engagement along the HIV care continuum among African American/black and Hispanic persons living with HIV in the United States: a qualitative exploration. Int J Equity Health. (2017) 16:54. doi: 10.1186/s12939-017-0549-3, PMID: 28340589 PMC5364619

[31] Harris RB Cormack DM Stanley J. Experience of racism and associations with unmet need and healthcare satisfaction: the 2011/12 adult New Zealand health survey. Aust N Z J Public Health. (2019) 43:75–80. doi: 10.1111/1753-6405.12835, PMID: 30296819

[32] Ahlberg BM Hamed S Bradby H Moberg C Thapar-Bjorkert S. “Just throw it behind you and just keep going”: emotional labor when ethnic minority healthcare staff encounter racism in healthcare. Front Sociol. (2022) 6, 1–11. doi: 10.3389/fsoc.2021.741202, PMID: 35097059 PMC8789661

[33] Cortis JD. Perceptions and experiences with nursing care: a study of Pakistani (Urdu) communities in the United Kingdom. J Transcultural Nurs: Official J Transcultural Nurs Society. (2000) 11:111–8. doi: 10.1177/104365960001100205, PMID: 11982043

[34] Kang C Tomkow L Farrington R. Access to primary health care for asylum seekers and refugees: a qualitative study of service user experiences in the UK. British J General Prac: J Royal College of General Practitioners. (2019) 69:e537–45. doi: 10.3399/bjgp19X701309, PMID: 30745354 PMC6617541

[35] Pérez-Urdiales I Goicolea I Sebastián MS Irazusta A Linander I. Sub-Saharan African immigrant women’s experiences of (lack of) access to appropriate healthcare in the public health system in the Basque Country, Spain. Int J Equity Health. (2019) 18:59. doi: 10.1186/s12939-019-0958-6, PMID: 31014337 PMC6480650

[36] Möschel M. Race in mainland European legal analysis: towards a European critical race theory. Ethn Racial Stud. (2011) 34:1648–64. doi: 10.1080/01419870.2011.566623, PMID: 39699680

[37] Borevi K. From multi-culturalism to assimilation? Swedish integration policy from a European perspective. Statsvetensk Tidskr. (2011) 113:47–56.

[38] Bradby H Thapar-Björkert S Hamed S Ahlberg BM. Undoing the unspeakable: researching racism in Swedish healthcare using a participatory process to build dialogue. Health Res Policy Syst. (2019) 17:43. doi: 10.1186/s12961-019-0443-0, PMID: 31014361 PMC6480694

[39] Bojadzijev M Braun K Opratko B Liebig M Heiter A. Entsolidarisierung und Rassismus. Forschungs-Interventions-Cluster Solidarität im Wandel? Berlin: Berliner Institut für empirische Integrations- und Migrationsforschung (2017).

[40] Lentin A. Europe and the silence about race. Eur J Soc Theory. (2008) 11:487–503. doi: 10.1177/1368431008097008

[41] Salem S. Thompson V. (2016). Old racisms, new masks: on the continuing discontinuities of racism and the erasure of race in European contexts. Nineteen sixty nine: an ethnic studies journal 3. Available at: https://escholarship.org/uc/item/98p8q169 (Accessed December 30, 2024).

[42] Spallek J Razum O. Migration und Gesundheit In: Richter M Hurrelmann K, editors. Soziologie von Gesundheit und Krankheit. Heidelberg: Springer VS (2016). 153–66.

[43] Yeboah A. Rassismus und psychische Gesundheit in Deutschland In: Fereidooni K Meral E, editors. Rassismuskritik und Widerstandsformen. Heidelberg: Springer VS (2017). 143–61.

[44] Velho A. (2010). Un/Tiefen der Macht: Auswirkungen von Rassismuserfahrungen auf die Gesundheit, das Befinden und die Subjektivität. Ansätze für eine reflexive Berufspraxis. Antidiskriminierungsstelle für Menschen mit Migrationshintergrund (AMIGRA).

[45] Gerlach H Becker N Fuchs A Wollny A Abholz H-H. Diskriminierung von Schwarzen aufgrund ihrer Hautfarbe? Ergebnisse von Focusgruppendiskussionen mit Betroffenen im deutschen Gesundheitswesen. Gesundheitswesen. (2008) 70:47–53. doi: 10.1055/s-2007-1022524, PMID: 18273763

[46] Gerlach H Becker N Abholz H-H. Welche Erfahrungen haben deutsche Hausärzte mit Patienten mit Migrationshintergrund? Ergebnisse einer Fokusgruppendiskussion mit Hausärzten. Z Allgemeinmed. (2008) 84:428–35. doi: 10.1055/s-0028-1087184

[47] Gerlach H. Abholz H.-H. Koc G. Yilmaz M. Becker N. (2012). “Ich möchte als Migrant auch nicht anders behandelt werden.” Fokusgruppen zu Erfahrungen von Patienten mit Migrationshintergrund aus der Türkei. Zeitschrift für Allgemeinmedizin 88, 77–85.

[48] Nowak AC Hornberg C. Erfahrungen von Menschen mit Fluchtgeschichte bei der Inanspruchnahme der Gesundheitsversorgung in Deutschland – Erkenntnisse einer qualitativen Studie. Bundesgesundheitsbl Gesundheitsforsch Gesundheitsschutz. (2023) 66:1117–25. doi: 10.1007/s00103-022-03614-y, PMID: 36445459 PMC10539425

[49] Spura A Kleinke M Robra B Ladebeck N. Wie erleben Asylsuchende den Zugang zu medizinischer Versorgung? Bundesgesundheitsbl Gesundheitsforsch Gesundheitsschutz. (2017) 60:462–70. doi: 10.1007/s00103-017-2525-x, PMID: 28229173

[50] Schödwell S Savin M Lauke A Abels I Abdel-Fatah D Penka S. Erratum zu: Strukturelle Diskriminierung und Rassismus in der Krankenhausversorgung: die Rolle ökonomischer Rahmenbedingungen in der interkulturellen Öffnung. Bundesgesundheitsbl Gesundheitsforsch Gesundheitsschutz. (2023) 66:101–2. doi: 10.1007/s00103-022-03624-w, PMID: 36472644 PMC9831949

[51] Savitt T. The use of blacks for medical experimentation and demonstration in the old south. J South Hist. (1982) 48:331–48. doi: 10.2307/2207450, PMID: 11645888

[52] Washington H. Medical apartheid: The dark history of medical experimentation on black Americans from colonial times to the present. New York: Doubleday (2007).

[53] Bauche M. Medizin und Herrschaft: Malariabekämpfung in Kamerun, Ostafrika und Ostfriesland (1890–1919). Frankfurt: Campus (2017).

[54] Siouti I Tuider E von Unger H Yildiz E. Othering in der Postmigrantischen Gesellschaft. Herausforderungen und Konsequenzen für die Forschungspraxis. Bielefeld: Transcript (2022).

[55] Smith L. Decolonizing methodologies: Research and indigenous peoples. London: Zed Books (2021).

[56] Zuberi T Bonilla-Silva E. White logic, white methods: Racism and methodology. Lanham, MD: Rowman & Littlefield (2008).

[57] von Unger H Scott P Odukoya D. Constructing im/migrants and ethnic minority groups as “carriers of disease”: power effects of categorization practices in tuberculosis health reporting in the UK and Germany. Ethnicities. (2019) 19:518–34. doi: 10.1177/1468796819833426

[58] Aljadeeah S. Are refugees (really) a hard-to-survey group? Fieldwork experience with Syrian refugees in Germany. J Refug Stud. (2022) 35:1405–9. doi: 10.1093/jrs/feac025

[59] Wallerstein N Duran B Oetzel J Minkler M. On community-based participatory research In: Wallerstein N Duran B Oetzel J Minkler M, editors. Community-based participatory research for health: Advancing social and health equity. Hoboken, NJ: Jossey-Bass (2018). 17–29.

[60] Dotson K. Conceptualizing epistemic oppression. Soc Epistemol. (2014) 28:115–38. doi: 10.1080/02691728.2013.782585

[61] Spivak G. Can the subaltern speak? In: Nelson C Grossberg L, editors. Marxism and the interpretation of culture. New York: Macmillan (1988). 24–8.

[62] Hall B Godrie B Heck I. Knowledge democracy and epistemic in/justice: reflections on a conversation. Canadian J Action Res. (2020) 21:27–45. doi: 10.33524/cjar.v21i1.516

[63] Wallerstein N. Commentary on community-based participatory research and community engaged research in health for journal of participatory research methods. J Participatory Res Methods. (2020) 1:1–8. doi: 10.35844/001c.13274

[64] Israel BA Schulz AJ Parker EA Becker AB. Review of community-based research: assessing partnership approaches to improve public health. Annu Rev Public Health. (1998) 19:173–202. doi: 10.1146/annurev.publhealth.19.1.173, PMID: 9611617

[65] Minkler M Garcia AP Rubin V Wallerstein N. Community-based participatory research: A strategy for building healthy communities and promoting health through policy change. Oakland: Policy Link (2012).

[66] Spivak G. The Rani of Sirmur: an essay in reading the archives. History and Theory. (1985) 24:247–72. doi: 10.2307/2505169

[67] Roche B Guta A Flicker S. Peer research in action I: Models of practice, Community-based research working paper series. Toronto: Wellesley Institute (2010).

[68] Bär G Kasberg A Geers S Clar C. Fokusgruppen in der partizipativen Forschung In: Hartung S Wihofszky P Wright MT, editors. Partizipative Forschung. Wiesbaden: Springer Fachmedien (2020). 207–32.

[69] Israel B Coombe C Cheezum R Schulz A Mcgranaghan R Lichtenstein R. Community-based participatory research: a capacity-building approach for policy advocacy aimed at eliminating health disparities. Am J Public Health. (2010) 100:2094–102. doi: 10.2105/AJPH.2009.170506, PMID: 20864728 PMC2951933

[70] Krueger RA Casey MA. Focus groups: A practical guide for applied research. Thousand Oaks, CA: SAGE (2015).

[71] Flicker S Nixon SA. The DEPICT model for participatory qualitative health promotion research analysis piloted in Canada, Zambia and South Africa. Health Promot Int. (2015) 30:616–24. doi: 10.1093/heapro/dat093, PMID: 24418997 PMC4542917

[72] Russo J. Survivor-controlled research: a new foundation for thinking about psychiatry and mental health. Forum qualitative Sozialforschung / forum. Qual Res. (2012) 13, 1–29. doi: 10.17169/fqs-13.1.1790

[73] von Unger H. (2022). “Diversifizierung, Reflexivität und Partizipation: Strategien gegen Ver-Anderung in der Forschung,” in Othering in der postmigrantischen Gesellschaft: Herausforderungen und Konsequenzen für die Forschungspraxis, eds. Siouti I. Spies T. Tuider E.., Unger H. von Yildiz E. (Bielefeld: transcript), 85–106.

[74] Said EW. Orientalism. New York: Routledge and Kegan Paul (1978).

[75] Hall S. Rassismus als ideologischer Diskurs In: Räthzel N, editor. Theorien über Rassismus. Hamburg: Argument (2000). 7–16.

[76] Benkert R Peters RM. African American women’s coping with health care prejudice. West J Nurs Res. (2005) 27:863–89. doi: 10.1177/0193945905278588, PMID: 16275704

[77] Collins PH. Black feminist thought: Knowledge, consciousness, and the politics of empowerment. New York: Routledge (2000).

[78] Attia I. Rassismus (nicht) beim Namen nennen. APuZ (Aus Politik und Zeitgeschichte). (2014) 64:8–14.

[79] Lafaut D Coene G. “I was trying to speak to their human side”: coping responses of Belgium’s undocumented migrants to barriers in health-care access. Int J Migration, Health and Social Care. (2020) 16:253–67. doi: 10.1108/IJMHSC-05-2019-0051

[80] Scott P. Black African asylum seekers’ experiences of health care access in an eastern German state. Int J Migration, Health and Social Care. (2014) 10:134–47. doi: 10.1108/IJMHSC-11-2013-0043

[81] Hall S. Rassismus und kulturelle Identität, Ausgewählte Schriften 2. Hamburg: Argument (1994).

[82] Lazaridou FB Schubert SJ Ringeisen T Kaminski J Heinz A Kluge U. Racism and psychosis: an umbrella review and qualitative analysis of the mental health consequences of racism. Eur Arch Psychiatry Clin Neurosci. (2023) 273:1009–22. doi: 10.1007/s00406-022-01468-8, PMID: 36001139 PMC9400567

[83] Paradies Y. Racism and health In: Quah SR and Cockerham WC, editors. The interna2onal encyclopedia of public health, *2nd ed*. Oxford: Academic Press (2017), 249–59.

[84] Musa D Schulz R Harris R Silverman M Thomas SB. Trust in the health care system and the use of preventive health services by older black and white adults. Am J Public Health. (2009) 99:1293–9. doi: 10.2105/AJPH.2007.123927, PMID: 18923129 PMC2696665

[85] Powell W Richmond J Mohottige D Yen I Joslyn A Corbie-Smith G. Medical mistrust, racism, and delays in preventive health screening among African-American men. Behav Med. (2019) 45:102–17. doi: 10.1080/08964289.2019.1585327, PMID: 31343960 PMC8620213

[86] Sabbah W Gireesh A Chari M Delgado-Angulo EK Bernabé E. Racial discrimination and uptake of dental services among American adults. Int J Environ Res Public Health. (2019) 16:1558. doi: 10.3390/ijerph16091558, PMID: 31060202 PMC6540199

[87] Wamala S Merlo J Boström G Hogstedt C. Perceived discrimination, socioeconomic disadvantage and refraining from seeking medical treatment in Sweden. J Epidemiol Community Health. (2007) 61:409–15. doi: 10.1136/jech.2006.049999, PMID: 17435207 PMC2465685

[88] Ha KN Al-Samarai NL Mysorekar S. Einleitung In: Ha KN Al-Samarai NL Mysorekar S, editors. re/visionen. Postkoloniale Perspektiven von People of Color auf Rassismus, Kulturpolitik und Widerstand in Deutschland. Münster: Unrast (2007). 9–23.

[89] Dotson K. Tracking epistemic violence, tracking practices of silencing. Hypatia. (2011) 26:236–57. doi: 10.1111/j.1527-2001.2011.01177.x

[90] Sue DW Capodilupo CM Torino GC Bucceri JM Holder AM Nadal KL. Racial microaggressions in everyday life: implications for clinical practice. Am Psychol. (2007) 62:271–86. doi: 10.1037/0003-066X.62.4.271, PMID: 17516773

[91] Hamed S Bradby H. Racism and racialisation in healthcare settings In: Petersen A, editor. Handbook on the sociology of health and medicine. Cheltenham: Edward Elgar (2023). 354–77.

[92] Hall B. From margins to center: the development and purpose of participatory action research. Am Sociol. (1992) 23:15–28. doi: 10.1007/BF02691928

[93] Haraway D. Situated knowledges: the science question in feminism and the privilege of partial perspective. Fem Stud. (1988) 14:575–99. doi: 10.2307/3178066

